# Green tea inhibited the elimination of nephro-cardiovascular toxins and deteriorated the renal function in rats with renal failure

**DOI:** 10.1038/srep16226

**Published:** 2015-11-10

**Authors:** Yu-Hsuan Peng, Douglas H. Sweet, Shiuan-Pey Lin, Chung-Ping Yu, Pei-Dawn Lee Chao, Yu-Chi Hou

**Affiliations:** 1 School of Pharmacy, China Medical University, Taichung, Taiwan, R.O.C; 2 Department of Pharmaceutics, Virginia Commonwealth University, Richmond, U.S.A; 3 Department of Pharmacy, China Medical University Hospital, Taichung, Taiwan, R.O.C; 4 Department of Medical Research, China Medical University Hospital, Taichung, Taiwan, R.O.C

## Abstract

Chronic kidney disease (CKD) is a major health problem worldwide. Indoxyl sulfate (IS) and *p*-cresyl sulfate (PCS) are highly protein-bound nephro-cardiovascular toxins, which are not efficiently removed through hemodialysis. The renal excretions of IS and PCS were mediated by organic anion transporters (OATs) such as OAT1 and OAT3. Green tea (GT) is a popular beverage containing plenty of catechins. Previous pharmacokinetic studies of teas have shown that the major molecules present in the bloodstream are the glucuronides/sulfates of tea catechins, which are putative substrates of OATs. Here we demonstrated that GT ingestion significantly elevated the systemic exposures of endogenous IS and PCS in rats with chronic renal failure (CRF). More importantly, GT also significantly increased the levels of serum creatinine (Cr) and blood urea nitrogen (BUN) in CRF rats. Mechanism studies indicated that the serum metabolites of GT (GTM) inhibited the uptake transporting functions of OAT1 and OAT3. In conclusion, GT inhibited the elimination of nephro-cardiovascular toxins such as IS and PCS, and deteriorated the renal function in CRF rats.

Chronic kidney disease (CKD) is affecting the health of more and more people worldwide^1^. The main feature at the end stage of CKD is the accumulation of endogenous uremic toxins^2^,^3^, among which indoxyl sulfate (IS) and *p*-cresyl sulfate (PCS) are highly protein-bound and cannot be efficiently removed through hemodialysis^4^. Moreover, the elevated serum levels of IS and PCS were associated with the progression of CKD and cardiovascular diseases (CVD) as well as all-cause mortality^5^,^6^,^7^. Owing to the acidic properties, IS and PCS are existing as anions under physiological pH in the systemic circulation, and the uptake transports of IS and PCS across the cell membranes of renal proximal tubules were mediated by organic anion transporters (OATs) such as OAT1 and OAT3^8^,^9^,^10^.

Habitual consumption of teas, leaves and buds of *Cammellia sinensis*, has long been considered to have many health benefits such as prevention of CVD and cancers^11^,^12^. Green tea (GT) is one of the most popular teas containing plenty of catechins such as (−)-epicatechin (EC), (−)-epigallocatechin (EGC), (−)-epicatechin-3-gallate (ECG) and (−)-epigallocatechin-3-gallate (EGCG), which account for up to 30% of the dry leaf weight^13^. Previous studies on tea catechin pharmacokinetics have reported that EC glucuronides/sulfates and EGC glucuronides/sulfates were the major molecules in the bloodstream^14^,^15^,^16^. Given the anionic nature of glucuronides/sulfates under physiological pH, these conjugated metabolites of tea catechins are putative substrates of OATs^17^. We therefore hypothesized that the serum metabolites of GT (GTM) might inhibit the uptake transport of IS and PCS across the cell membrane of renal proximal tubule mediated by OAT1 and OAT3, which in turn would lead to elevated blood levels of IS and PCS in CKD patients with lower expressions of OATs^18^,^19^, and consequently promote the progression of CKD and CVD.

Although GT has been found to show renal and cardiovascular protective effects in healthy subjects^20^,^21^,^22^, till now whether GT likely benefits CKD patients remains unknown. Previous studies have reported that adenine-induced chronic renal failure (CRF) rats showed lower protein expressions of OAT1 and OAT3 along with the decreased clearance of IS and PCS^19^,^23^. Hence, a CRF rat model induced with adenine was adopted in this study to investigate the effect of GT on the serum levels of endogenous IS and PCS. Moreover, the effect of GT on the renal function of CRF rats was also evaluated. Furthermore, cell models were used to verify the involvement of OAT1 and OAT3 in the mechanism.

## Results

### Quantitation of tea catechins in GT infusion

**Figure 1:**
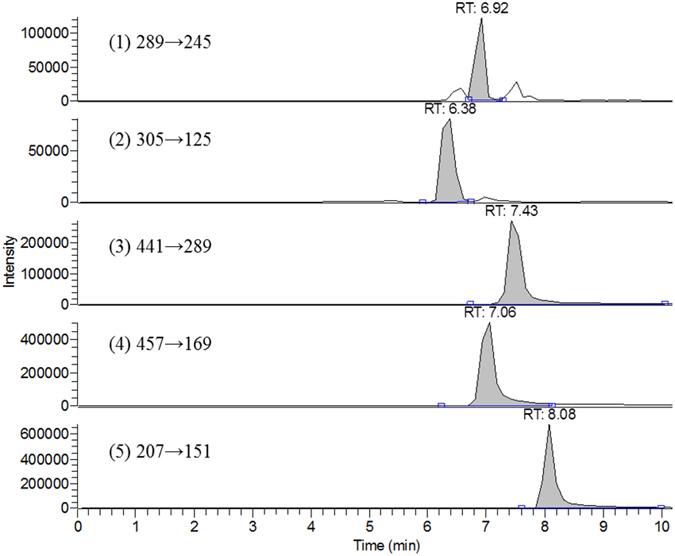
LC-MS/MS chromatograms of EC (1), EGC (2), ECG (3), EGCG (4) and 6,7-DMC (5, internal standard) in GT infusion.

GT infusion was analyzed by LC-MS/MS and the chromatograms were shown in Fig. 1. The quantitation results showed that the concentrations of EC, EGC, ECG and EGCG were 966.7, 919.9, 739.7 and 1960.1 μg/mL (3.33, 3.17, 2.55 and 6.75 mM), respectively, in the GT infusion.

### Characterization of the serum metabolites of GT (GTM)

**Figure 2:**
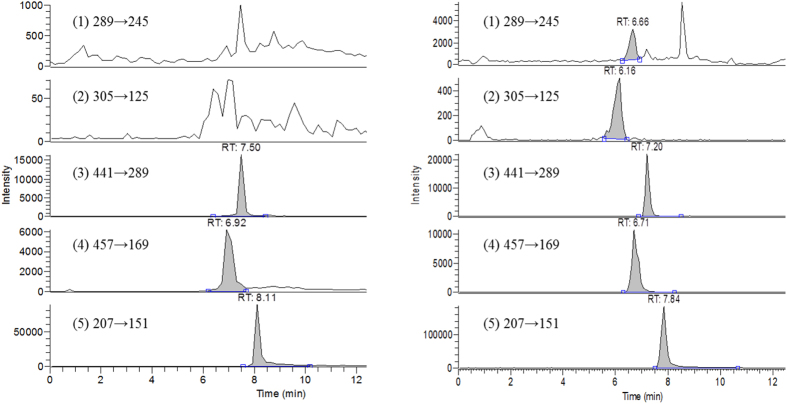
LC-MS/MS chromatograms of EC (1), EGC (2), ECG (3), EGCG (4) and 6,7-DMC (5, internal standard) in serum before (left) and after treatment with glucuronidase/sulfatase (right).

For mimicking the molecules interacting with OAT1 and OAT3 located on the membrane of renal proximal tubular cells, GTM was prepared from rats after receiving GT infusion. Characterization of GTM indicated that the concentrations of the free forms of EC, EGC, ECG and EGCG were 0.86, 2.17, 0.06 and 0.13 μM, respectively. After hydrolysis with sulfatase/glucuronidase, the peaks of EC, EGC, ECG and EGCG were all enhanced, in particular EC and EGC as shown in Fig. 2. The concentrations of free form and the sulfates/glucuronides of each tea catechin in GTM are listed in Table 1, showing that the major molecules in GTM were the sulfates/glucuronides of EC and EGC. The concentrations of the sulfates/glucuronides of EC, EGC, ECG and EGCG were 32.47, 33.29, 0.13 and 0.40 μM, respectively, which have been obtained by subtracting the free form concentrations from those after hydrolysis.

### Quantitation of IS and PCS in serum

The analytical methods of IS and PCS were established and validated in this study. Good linear relationships existed in the concentration ranges of 0.4–25.0 μg/mL (Y = 0.246 X + 0.006, r = 0.9999) for IS and 0.3–20.0 μg/mL (Y = 0.045 X + 0.010, r = 0.9996) for PCS in serum. Validation of the analytical methods for IS indicated that the coefficients of variation (CVs) of intraday and interday analysis were below 6.4% and 7.6%, and the relative errors (R.E.) were below 12.1% and 9.9%, respectively. The CVs of PCS of intraday and interday analysis were below 17.0% and 12.6%, and the R.E. were below 10.9% and 7.8%. The recoveries of IS at 0.8, 3.1 and 12.5 μg/mL from serum were 88, 101 and 100%, and those of PCS at 0.6, 2.5 and 10.0 μg/mL were 104, 103 and 105%. The LLOQ of IS and PCS were 0.4 and 0.3 μg/mL, and LOD were 0.01 and 0.04 μg/mL.

### Establishment of CRF model and effect of GT on the serum levels of IS and PCS in CRF rats

**Figure 3:**
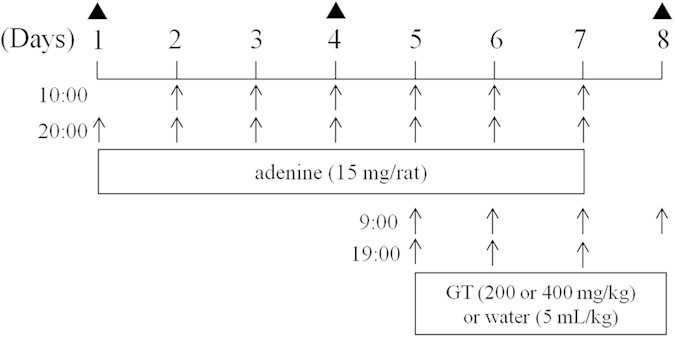
Scheduled time of blood collection (▲) and dosing time of adenine and GT (↑).

**Figure 4:**
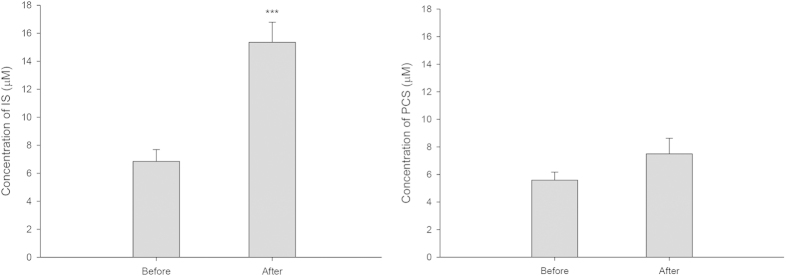
Mean (±S.E.) serum IS (left) and PCS (right) concentrations in 28 rats before and after administration of seven doses of adenine (15 mg/rat).***P < 0.001.

**Figure 5:**
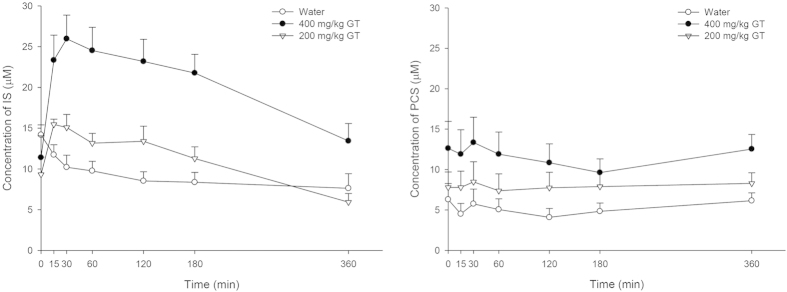
Mean (±S.E.) serum concentration - time profiles of endogenous IS (left) and PCS (right) after administration of the 7^th^ dose of 400 mg/kg of GT (•, n = 10), 200 mg/kg of GT (▽, n = 8) and water (○, n = 10) in CRF rats.

The dosing times of adenine and GT as well as scheduled time of blood collection are summarized in Fig. 3. After administration of five consecutive doses of adenine, the mean serum IS level in 28 rats was significantly elevated from 6.85 to 15.35 μM (P < 0.001), and PCS level was increased from 5.59 to 7.49 μM (P = 0.14) on day 4 as shown in Fig. 4. The serum profiles of endogenous IS and PCS in CRF rats following oral administration of GT at 200 and 400 mg/kg are shown in Fig. 5. The profiles clearly showed that ingestion of GT dose-dependently increased the serum levels of IS and PCS. The pharmacokinetic parameters of IS and PCS are listed in Table 2. The results showed that ingestion of 400 mg/kg of GT significantly increased the AUC_0-360_ of IS and PCS by 139.2% and 118.4%, respectively, and elevated C_max_ of IS by 123.3%, whereas the C_max_ of PCS was not affected. In contrast, when GT was given at a lower dose of 200 mg/kg, the profiles of IS and PCS were higher than the control curves, however, the differences of C_max_ and AUC_0-360_ did not reach statistical significance.

### Effect of GT on the renal function of CRF rats

After administration of five consecutive doses of adenine, the mean Cr of 14 rats was significantly elevated from 0.30 to 0.58 mg/dL, and BUN was increased from 21.0 to 60.1 mg/dL on day 4. The concentrations of Cr and BUN in CRF rats after giving the 7^th^ dose of GT and water are shown in Table 3. The mean Cr and BUN in GT group were 0.79 and 55.1 mg/dL, respectively, which were significantly higher than those in water group (0.49 and 40.9 mg/dL).

### Effects of GTM on the uptake transport mediated by hOAT1 and hOAT3

**Figure 6:**
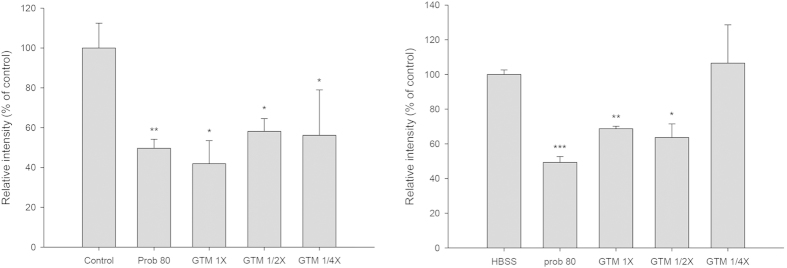
Effects of GTM (1, 1/2 and 1/4 -fold serum concentrations) and probenecid (Prob, 80 μM) on the intracellular accumulation of 6-CF in CHO-hOAT1 cells (left) and 5-CF in HEK293-hOAT3 cells (right). *P < 0.05, **P < 0.01 and ***P < 0.001.

The effects of GTM on the uptake activity of hOAT1 and hOAT3 are shown in Fig. 6. GTM at 1/4−, 1/2− and 1-fold serum concentrations significantly reduced the intracellular accumulation of 6-CF, an OAT1 substrate, by 43.9, 41.9 and 58.1%, respectively, when compared with blank serum specimen at corresponding concentrations. Likewise, GTM at 1/2− and 1-fold serum concentration significantly reduced the intracellular accumulation of 5-CF, an OAT3 substrate, by 36.4% and 31.3%, respectively, when compared to blank serum specimen at corresponding concentration. As positive control inhibitors of hOAT1 and hOAT3, probenecid (80 μM) significantly reduced the intracellular accumulation of 6-CF and 5-CF by 50.4 and 50.7%, respectively. These *in vitro* studies indicated that GTM significantly inhibited the uptake transport mediated by hOAT1 and hOAT3.

## Discussion

Quantitation results revealed that EGCG was the major tea catechin in GT infusion, whereas the major molecules in GTM were the sulfates/glucuronides of EC and EGC. These finding largely resonated with the results reported previously^16^. Based on these facts, we could infer that ECG and EGCG were feasibly hydrolyzed by esterase to form EC and EGC, respectively, which were then extensively metabolized by conjugation reactions to yield EC sulfates/glucuronides and EGC sulfates/glucuronides as the major molecules circulating in the bloodstream.

In regard to the quantitation of IS and PCS in serum, two HPLC methods using different internal standard and detection wavelengths have been developed and optimized in this study owing to the discrepant physicochemical properties between IS and PCS. Validation of the analytical methods confirmed that the precision, accuracy and recovery were satisfactory for the quantitation of IS and PCS in serum.

In clinical setting, CKD patients are often exposed to increased blood levels of endogenous uremic toxins such as IS and PCS, which are by-products of protein metabolism^24^,^25^. A recent study reported that the serum concentrations of IS and PCS in healthy subjects were 1.03 and 13.03 μM^26^. In contrast, for non-hemodialyzed stage 3–5 CKD patients the serum concentrations of IS and PCS were 17.45 and 73.47 μM, while in those CKD patients undergoing treatment with hemodialysis (stage 5D), they were 81.04 and 120.54 μM^26^. This highlights the fact that IS and PCS are highly protein bound (89% and 96%) and thus not efficiently removed through hemodialysis^27^,^28^,^29^,^30^. In regard to the relative abundance of IS and PCS in blood, the IS level was found consistently higher than PCS level in rats^31^,^32^,^33^, which appears opposite to that in humans. This discrepancy between rats and humans might stem from species difference in protein metabolism^23^,^27^,^34^.

In order to mimic the biological condition of CKD with lower expression of OATs, adenine was used to induce CRF in this study^19^,^23^,^35^,^36^. The serum levels of IS and PCS in our CRF rats had been elevated to 15.4 and 7.6 μM, which were largely close to those values in previous studies^31^,^32^,^33^. On other hand, the levels of serum Cr and BUN were significantly elevated, indicating that the renal function of rats was impaired by adenine^36^,^37^. After giving seven doses of GT to the CRF rats, the higher dose of GT at 400 mg/kg significantly increased the systemic exposures of IS and PCS, whereas the lower dose at 200 mg/kg did not result in significant increases, indicating that large quantity of GT inhibited the elimination of IS and PCS. Moreover, the levels of serum Cr and BUN were significantly increased by 400 mg/kg of GT, indicating that GT deteriorated the renal function of CRF rats, which might be accounted for by the increased systemic exposure of uremic toxins such as IS and PCS.

The uptake transports of IS and PCS across cell membrane were well known to be mediated by OAT1 and OAT3^8^,^9^. In regard to the magnitudes of influences, it was apparent that the effect of GT on the serum levels of IS was in a greater extent than PCS. It has been reported that the uptakes of IS by OAT1 and OAT3 were saturable with *K*_m_ values of 21 μM and 263 μM, respectively^38^. Regarding PCS, the *K*_m_ for the OAT1-mediated uptake was 128 μM, whereas saturation was not observed for OAT3-mediated transport up to 5 mM^39^. Given the lower *K*_m_ values of IS than PCS in both OAT1- and OAT3- mediated uptake, IS should be a substrate for OAT1 and OAT3 with lower capacity. Therefore, the uptake transports of IS mediated by OAT1 and OAT3, especially OAT1, were more easily to be saturated than those of PCS, which can account for the fact that GT elevated the serum levels of IS more than PCS.

In order to explore the mechanisms involved, GTM was prepared for mimicking the molecules interacting with renal OAT1 and OAT3^40^,^41^. Characterization of GTM demonstrated that the major molecules in the serum were EC glucuronides/sulfates and EGC glucuronides/sulfates, which echoed the finding in precedent studies^14^,^16^. Cell line studies showing that GTM exhibited inhibition on the uptake transport mediated by OAT1 and OAT3 could explain the increased serum levels of IS and PCS caused by GT. We speculated that these mechanisms associated with OAT1 and OAT3 in CRF rats could be extrapolated to CKD patients, because the distribution and function of OATs in rat kidney are similar to those in humans^42^,^43^,^44^.

Despite various health benefits of tea consumption to healthy humans^20^,^21^,^22^, the present study demonstrated that GT inhibited the elimination of nepro-vascular toxins such as IS and PCS, and deteriorated the renal function in CRF rats. Considering that high blood levels of IS and PCS increased oxidative stress, inflammatory mediators and chemokines in the body^45^, we suspect consuming large quantity of GT might result in promoting the progression of CKD and CVD in CKD patients. Moreover, owing to the inefficient removal of IS and PCS through hemodialysis, GT consumption might also exacerbate the pathologies of CKD and CVD in end-stage CKD patients even under hemodialysis treatment^5^,^6^,^7^. Therefore, we suggest that CKD patients should avoid drinking large quantity of tea beverages before benefits or risks to CKD patients assessed by future clinical studies. In conclusion, GT inhibited the elimination of nephro-cardiovascular toxins such as IS and PCS through inhibition on OAT1 and OAT3, and deteriorated the renal function in CRF rats.

## Materials and Methods

### Chemicals and reagents

GT was purchased from the market in Taichung, Taiwan. IS (purity 97%) was obtained from Alfa Aesar (Lancaster, UK). 6,7-dimethoxycoumarin (6,7-DMC, purity 98%) was supplied by Aldrich Chemical Co. (Milwaukee, WI, U.S.A.). EC (purity 90%), ECG (purity 98%), EGCG (purity 95%), formic acid, probenecid, phosphoric acid (glacial, 85%) and methyl-paraben were purchased from Sigma Chemical Co. (St. Louis, MO, U.S.A.). EGC (purity 92.7%) was obtained from ChromaDex, Inc. (Irvine, CA, U.S.A.). and ethyl acetate were LC grade and obtained from ECHO Chemical Co. (Miaoli Hsien, Taiwan). Acetonitrile was LC/MS grade and was purchased from Millinckrodt Baker, Inc. (Phillipsburg, NJ, U.S.A.). Fetal bovine serum was obtained from Biological Industries Inc. (Kibbutz, Beit Haemek, Israel). Penicillin-Streptomycin-Glutamine, Dulbecco’ s Modified Eagle Medium, trypsin/EDTA, Hank’s Balanced Salt Solution and 4-(2-hydroxyethyl)-1-piperazineethanesulfonic acid were purchased from Invitrogen (Carlsbad, CA, U.S.A.). Dulbecco’s Modified Eagle Medium F12 was obtained from Thermo Fisher Scientific Inc (Waltham, MA, U.S.A.). 6-Carboxyfluorescein was purchased from AAT Bioquest Inc. (Sunnyvale, CA, U.S.A.) and 5-carboxyfluorescein was obtained from Acros Organics (Geel, Belgium). Milli-Q plus water (Millipore, Bedford, MA, U.S.A.) was used for all preparations.

### Preparation and characterization of GT infusion

The infusion was produced by steeping either 2 or 4 g of GT in 50 mL of hot deionized water (98–100 °C) for 30 min. The infusion was filtered with gauze while hot to afford concentrations of 40 and 80 mg/mL, respectively, which was freshly administered to rats via gastric gavage.

For the characterization of GT infusion, after filtration of GT infusion with 0.2 μm RC15 filter (Sartorious, Goettingen Germany), 100 μL of the filtrate was mixed with 900 μL of methanol and centrifuged to remove the precipitate. The properly diluted infusion (50 μL) was combined with 50 μL of 6,7-DMC solution (2.0 μg/mL in methanol) as internal standard, and 5 μL was subject to LC-MS/MS analysis. The HPLC system included Accela 1250 pump and auto-sampler (Thermo Fisher Scientific Inc. U.S.A.). Chromatographic separation was achieved using a Phenomenex^**®**^ C18 analytical column (150 mm × 1.0 mm, 5 μm) with a prefilter. The mobile phase consisted of acetonitrile containing 0.01% formic acid (A) and water containing 0.01% formic acid (B) and programmed in a gradient manner as follows: A/B: 2/98 (0–2 min), 15/85 (4 min), 40/60 (6 min), 90/10 (8–10 min) and 2/98 (12 min). The flow rate was 0.2 mL/min. The column effluent was detected by H-ESI (heated-electrospray ionization) -II probe with Quantum Access MAX triple stage quadrupole (TSQ) mass spectrometer (Thermo Fisher Scientific Inc. U.S.A.). Nitrogen was used as sheath gas at 35 arbitrary units and auxiliary gas at 10 arbitrary units. The collision energy was set at −19/18 V, spray voltage at −3000/3000 V, capillary temperature at 350 °C, vaporizer temperature at 350 °C and tube lens offset at −80/88 V. The following mass transitions were used for selected reaction monitoring analysis (SRM): EC (289/245), EGC (305/125), ECG (441/289), EGCG (457/169), and 6,7-DMC (207/151). The ESI-MS spectra were recorded in negative ion mode for EC, EGC, ECG and EGCG and positive ion mode for 6,7-DMC.

### Animals

Male Sprague-Dawley rats (270–360 g) were purchased from National Laboratory Animal Center (Taipei, Taiwan) and housed in conditioned environment with 12-h light/dark cycles. Food and water were acquired *ad libitum* until 12 h prior to experiments. Rats were divided into two groups. The first experiment used 28 rats to determine the effect of GT on the serum levels of IS and PCS in CRF rats; the second experiment used 14 rats to evaluate the effect of GT on the renal function of CRF rats. All animal experiments were carried out in strict accordance with the recommendations by ‘‘The Guidebook for the Care and Use of Laboratory Animals (2002)’’ published by the Chinese Society of Animal Science, Taiwan, R.O.C. The experimental protocol had been reviewed and approved on 08/01/2014 (Permit Number: 103–126-N) by the Insititutional Animal Care and Use Committee of China Medical University, Taiwan, R.O.C.

### Establishment of CRF model and administration of GT infusion

CRF was induced via oral administration of adenine following previous studies, but with some modification^19^,^23^,^35^,^36^. Briefly, adenine suspended in 0.5% methylcellulose 400 (15 mg/mL) was orally administered to 28 rats via gastric gavage twice daily for seven consecutive doses (15 mg/rat) throughout the experimental period. On day 4, the serum levels of IS were determined. Upon confirming attenuated renal function by significant elevation of serum IS levels, the CRF rats were divided into three groups (8–10 rats in each group) with comparable IS levels. The first group received 400 mg/5 mL/kg of GT infusion twice daily for seven consecutive doses; the second group received 200 mg/5 mL/kg of GT twice daily for seven consecutive doses; and the third group received 5 mL/kg of water in parallel as control. On day 8, rats were administered the 7^th^ dose of GT at 9:00 am after overnight fast, and the blood samples were collected at 0, 15, 30, 60, 120, 180 and 360 min after dosing. The scheduled time of blood collection and administrations of adenine and GT are summarized in Fig. 3. At each sampling time, 0.5 mL of blood was withdrawn under isoflurane anesthesia. The blood samples were collected in microtubes and centrifuged at 10,000 g for 15 min to obtain serum, which was stored at −20 °C before analysis.

### Determination of the concentrations of IS and PCS in serum

For the determination of IS and PCS concentrations in serum, 50 μL of serum specimen was vortexed with 200 μL of methanol containing 10 μg/mL of 6,7-DMC or 100 μg/mL of methylparaben as internal standards, respectively, and centrifuged to remove the precipitate, then a 20 μL aliquot was subject to HPLC analysis.

For calibrator preparations, 50 μL of serum was spiked with a series of concentrations of IS and PCS to afford concentration ranges of 0.4–25.0 μg/mL (1.8–117.3 μM) and 0.3–20.0 μg/mL (1.7–106.3 μM), respectively. The later procedure followed that described above for serum samples. The calibration curves were drawn by linear regression of the peak area ratios (IS or PCS to internal standard) against the known concentrations of IS and PCS, respectively.

The HPLC apparatus included a pump (LC-10AT, Shimadzu, Kyoto, Japan), fluorescence detector (RF-20A, Shimadzu, Kyoto, Japan) and automatic injector (SIL-10AF, Shimadzu, Kyoto, Japan). The Apollo C18 (5 μm, 4.6 mm × 250 mm, Alltech, Deerfield, IL, USA) column was equipped with a guard column (4.6 × 50 mm, 5 μm) (GL Science Inc., Tokyo, Japan). The mobile phase consisted of acetonitrile (A)–0.1% phosphoric acid (B) and programmed in a gradient manner as follows: A/B: 15/85 (0–7 min), 30/70 (9–22 min) and 15/85 (24–35 min) for IS, and 16/84 (0–10 min), 30/70 (13–22 min) and 16/84 (24–36 min) for PCS. Detector settings were E_x_ 280 nm/E_m_ 375 nm for IS and E_x_ 214 nm/E_m_ 306 nm for PCS. The flow rates were 1.0 mL/min.

### Effect of GT on Cr and BUN in CRF rats

In another experiment, the same animal protocol mentioned above was employed, and the concentrations of Cr and BUN were determined before adenine treatment (Day 0), before GT dosing (Day 4) and after 7^th^ dose of GT (Day 8). Upon confirming attenuated renal function by significant elevation of Cr and BUN on day 4, the CRF rats were divided into two groups (7 rats per group) with comparable Cr levels. The first group received 400 mg/5 mL/kg of GT twice daily for seven consecutive doses; the second group received 5 mL/kg of water in parallel as control. On day 8, rats were administered the 7^th^ dose of GT and water after overnight fast, and the blood samples were collected 30 min later. Cr was determined by a kinetic alkaline picrate method in an ADVIA 2400 Chemistry System (Siemens Health Care Diagnostics, Inc. Tarrytown, NY, U.S.A.). BUN was assayed by the urease/glutamate dehydrogenase coupled-enzyme reaction using the same analyser.

### Cell line and culture conditions

Construction of the stably transfected cell lines used for transport studies, Chinese hamster ovary (CHO) cells expressing hOAT1 (CHO-hOAT1), human embryonic kidney 293 (HEK) cells expressing hOAT3 (HEK-hOAT3) and their corresponding empty vector-transfected control cell lines, was described previously^40^,^41^. CHO cells were maintained at 37 °C with 5% CO_2_ in DMEM F-12 media (Thermo Fisher Scientific Inc., USA) containing 10% serum, 1% penicillin/streptomycin and 1 mg/mL G418. HEK cells were maintained at 37 °C with 5% CO_2_ in DMEM high glucose media (Thermo Fisher Scientific Inc., USA) containing 10% serum, 1% penicillin/streptomycin and 50 μg/mL hygromycin B. Cells were cultured in Poly-D-Lysine coated dishes.

### Preparation and characterization of the serum metabolites of GT (GTM)

In order to mimic the molecules interacting with OATs *in vivo*, GTM were prepared from rats and characterized. Briefly, GT infusion (800 mg/10 mL/kg) was orally administered to rats fasted overnight. Blood was collected at 15 min after dosing of GT infusion. After coagulation, the serum was collected and vortexed with 3-fold methanol. Following centrifugation at 10,000 g for 15 min, the supernatant was concentrated in a rotatory evaporator under vacuum to dryness. An appropriate volume of water was added to the residue yielding a solution with 10-fold serum concentration, which was divided into aliquots and stored at −80 °C for later use.

A portion of GTM was characterized following a previous method with some modifications^16^,^46^. Briefly, 100 μL of serum sample was mixed with 50 μL of sulfatase (containing 1000 units/mL of sulfatase and 39,861 units/ml of ß-glucuronidase), 50 μL of ascorbic acid (200 mg/mL) and incubated at 37 °C for 45 min under anaerobic condition. After hydrolysis, the serum was added to 50 μL 0.1 N HCl and then partitioned with 250 μL of ethyl acetate (containing 100 ng/mL of 6,7-DMC as internal standard). The ethyl acetate layer was evaporated under N_2_ to dryness and reconstituted with an appropriate volume of mobile phase prior to LC-MS/MS analysis. The mobile phase consisted of acetonitrile (A) – water containing 0.1% formic acid (B) and programmed in a gradient manner as follows: A/B: 2/98 (0–2 min), 15/85 (4 min), 40/60 (6 min), 90/10 (8–10 min) and 2/98 (12 min). The flow rate was 0.2 mL/min.

### Effects of GTM on the uptake transport mediated by hOAT1 and hOAT3

CHO-hOAT1 and HEK-hOAT3 cells (1 × 10^5^ cells/well) were cultured in a 96-well plate. 6-Carboxyfluorescein (6-CF) and 5-carboxyfluorescein (5-CF) were used as probes for evaluating the effect of GTM on the activity of hOAT1 and hOAT3, respectively^47^,^48^. In addition, probenecid (80 μM) was used as a positive control for inhibition of hOAT1 and hOAT3^49^. After 24 h or 48 h incubation of CHO-hOAT1 or HEK-hOAT3, the medium was removed and washed three times with PBS buffer. Before the transport experiment, CHO-hOAT1 and HEK-hOAT3 cells were pre-incubated with test agents (GTM and probenecid) at 37 °C. After 30 min incubation, 6-CF or 5-CF was then added and incubated for another 5 min and 10 min, respectively. The plates were immediately placed on ice bath, and the supernatants were removed and the cells were washed three times with ice-cold PBS. Subsequently, 100 μL of 0.1% Triton X-100 was added to lyse the cells and the fluorescence was measured with excitation at 485 nm and emission at 528 nm. To quantitate the content of protein in each well, 10 μL of cell lysate was added to 200 μL of diluted protein assay reagent (Bio-Rad, Hercules, CA, U.S.A.) and the optical density was measured at 570 nm. The relative intracellular accumulation of 6-CF or 5-CF was calculated by comparing with that of controls after protein correction.

### Data analysis

The peak serum concentration (C_max_) was obtained from experimental observation. The area under the serum concentration - time curve (AUC_0-t_) was calculated using trapezoidal rule to the last point. The differences of IS and PCS among three groups were analyzed by using one-way ANOVA, while the difference of Cr and BUN between two groups was analyzed by using unpaired Student’s *t-*test, taking p < 0.05 as significant level. Unpaired Student’s *t-*test was also used for the analysis of *in vitro* assays.

## Additional Information

**How to cite this article**: Peng, Y.-H. *et al.* Green tea inhibited the elimination of nephro-cardiovascular toxins and deteriorated the renal function in rats with renal failure. *Sci. Rep.* **5**, 16226; doi: 10.1038/srep16226 (2015).

## Figures and Tables

**Table 1:** Concentration (μM) of free form and the sulfates/glucuronides (S/G) of tea catechins in GTM.

Catechins	EC	EGC	ECG	EGCG
free form	0.86	2.17	0.06	0.13
S/G	32.47	33.29	0.13	0.40

**Table 2:** Effect of GT on C_max_ and AUC _0-360_ of IS and PCS in CRF rats.

Treatments	IS		PCS	
	C_max_	AUC _0-360_	C_max_	AUC _0-360_
Water	12.5 ± 1.4^a^	2957.8 ± 434.4^a^	7.8 ± 1.6	1767.4 ± 350.2^a^
GT 200 mg/kg	17.1 ± 1.4^a^	3747.4 ± 421.9^a^	10.1 ± 2.2	2737.3 ± 589.2^a,b^
GT 400 mg/kg	28.0 ± 2.7^b^	7074.7 ± 776.7^b^	10.0 ± 3.2	3859.3 ± 659.82 ^b^
	(+123.3%)	(+139.2%)		(+118.4%)

^a,b^Significant difference at P < 0.05 between means are denoted by different superscripts.

Data expressed as mean ± S.E.

C_max_ (μM): maximum plasma concentration.

AUC _0-360_ (μmol·min/L): the area under concentration-time curve from 0 to 360 min.

**Table 3:** Effect of GT on Cr and BUN (mg/dL) in 14 rats (7 rats/group).

	Cr		BUN	
**Treatments**	Water	GT	Water	GT
Day 0	0.30 ± 0.0	0.30 ± 0.0	21.1 ± 0.9	20.9 ± 1.2
Day 4	0.59 ± 0.0	0.57 ± 0.0	67.0 ± 6.3	53.1 ± 2.2
Day 8	0.49 ± 0.0	0.79 ± 0.1^*^	40.9 ± 3.3	55.1 ± 5.7^**^

^*^P < 0.05 compared to water group.

Day 0: before adenine treatment.

Day 4: before GT dosing.

Day 8: after 7^th^ dose of GT and water.

Data expressed as mean ± S.E.
